# Spectrum-Effect Relationships as a Systematic Approach to Traditional Chinese Medicine Research: Current Status and Future Perspectives

**DOI:** 10.3390/molecules191117897

**Published:** 2014-11-04

**Authors:** Guan-Ling Xu, Meng Xie, Xiao-Yan Yang, Yan Song, Cheng Yan, Yue Yang, Xia Zhang, Zi-Zhen Liu, Yu-Xin Tian, Yan Wang, Rui Jiang, Wei-Rui Liu, Xiao-Hong Wang, Gai-Mei She

**Affiliations:** 1 School of Chinese Pharmacy, Beijing University of Chinese Medicine, Beijing 100102, China; E-Mails: xuguanling4004@163.com (G.-L.X.); xiemeng0819@163.com (M.X.); yaner1888@163.com (X.-Y.Y.); 13269650940@163.com (C.Y.); 18311242015@163.com (Y.Y); zhangxia0561@126.com (X.Z.); lzz332@126.com (Z.-Z.L.); tianyuxin1216@163.com (Y.-X.T.); 18739908461@163.com (Y.W.); jiangrui54264@126.com (R.J.); liuweirui2012@126.com (W.-R.L.); bucm_pharm@126.com (X.-H.W.); 2 Pharmacy College, Ningxia Medical University, Ningxia 750000, China; E-Mail: songyan200714@163.com

**Keywords:** spectrum-effect relationships, traditional Chinese medicine, Chinese herbal formulas, fingerprints, drug-containing serum

## Abstract

Component fingerprints are a recognized method used worldwide to evaluate the quality of traditional Chinese medicines (TCMs). To foster the strengths and circumvent the weaknesses of the fingerprint technique in TCM, spectrum-effect relationships would complementarily clarify the nature of pharmacodynamic effects in the practice of TCM. The application of the spectrum-effect relationship method is crucial for understanding and interpreting TCM development, especially in the view of the trends towards TCM modernization and standardization. The basic requirement for using this method is in-depth knowledge of the active material basis and mechanisms of action. It is a novel and effective approach to study TCMs and great progress has been made, but to make it more accurate for TCM research purposes, more efforts are needed. In this review, the authors summarize the current knowledge about the spectrum-effect relationship method, including the fingerprint methods, pharmacodynamics studies and the methods of establishing relationships between the fingerprints and pharmacodynamics. Some speculation regarding future perspectives for spectrum-effect relationship approaches in TCM modernization and standardization are also proposed.

## 1. Introduction

Traditional Chinese medicines (TCMs) have been performing an increasingly important role in protecting health and controlling disease in China for thousands of years [1]. Based on their long history of clinical use and sound effects in the treatment of numerous diseases, especially chronic diseases, TCMs are widely accepted and used by billions of people around the world. Compared to chemical drugs TCMs have multi-target and multi-component characteristics when treating a disease. That means to say, the healing efficacy of a TCM depends on the combined action of multiple components because it usually contains a lot of ingredients. This can cause many difficulties to control the quality and in the search for the effective substance(s) of TCMs. Therefore, numerous researchers have devoted themselves to devising various means to solve these problems [2]. As a hotspot of TCM studies, spectrum-effect relationship method research has gradually drawn extensive attention and has been supposed to provide a way to clarify the active materials of TCMs and control TCM quality.

The term “spectrum-effect relationship” refers to linking the TCM fingerprint peaks with specific pharmacodynamic data to form a relationship, and then using this relationship to look for the effective materials in Chinese medicines and formulate control standards to reflect their internal quality. The spectrum-effect relationship method is developed based on the fingerprint technology. To overcome the shortcomings of fingerprints, it uses data processing technology to analyze the weight coefficients of components, monitoring efficacy despite changes of the peaks and their areas in the fingerprint [3]. This is where biochemistry, molecular biology, and cell biology are invaluable in establishing quantifiable and reproducible assays [4]. Thus, correlated peaks can help researchers further reveal the effective TCM material basis and control TCM quality. Being an interdisciplinary and cutting-edge science, the spectrum-effect relationship method is a technology integrating Chinese medicinal chemistry, analytical chemistry, the pharmacodynamics of TCMs and chemometrics, so it is considered a more accurate way to study TCM active materials.

**Figure 1:**
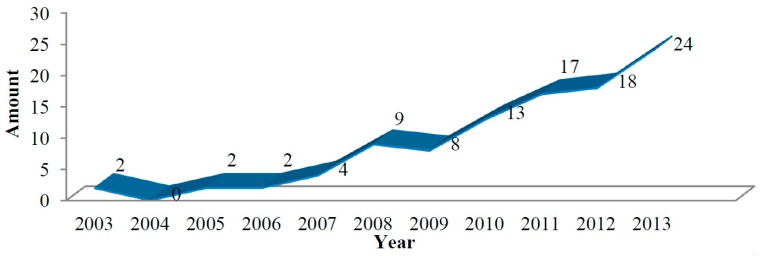
Line chart showing the total amount of research articles on the TCM spectrum-effect relationship approach published from 2003 to 2013.

The concept of the spectrum (fingerprint)-effect relationship was a brand new way of thinking at the frontier of the modernization of TCMs proposed by TCM researchers. It could reflect the real and comprehensive pharmacological information of active constituents. It was also more adequate for controlling the quality of TCMs. It has thus become a hotspot in TCM studies. The amount of published research work related to spectrum-effect relationship studies has been displaying a rising trend year after year. Figure 1 shows that studies on spectrum-effect relationships started to appear around 2003, and the research achievements in the area have been increasing in the last 10 years, with several articles pertaining to research on TCMs having been published in the last 8 years in Science Citation Index-cited journals such as *The Journal of Ethnopharmacology*, *Analytica Chimica Acta* and *Journal of Chromatography B*, *etc.* In the field of TCM research, there are 34 projects funded by National Natural Science Foundation from the year 2006. Moreover, more and more reviews have focused on spectrum-effect relationships and it is already widely applied in the quality control of TCM preparations in China. 

Based on the evolution, current research status and future perspectives, the purpose of this review is to summarize and merge the topics and research methods of the spectrum-effect relationship technique, so as to guide researchers to master this technology and encourage further research on TCMs.

## 2. The Evolution of Spectrum-Effect Relationships

As early as 2000 the WHO stated the following: “Research on traditional medicine safety and effectiveness of data are far from enough to meet the criteria that are needed to support its use around the world. It is because of a lack of adequate or accepted research methodology for evaluating traditional medicine, not only the deficiency of health care policies”. Containing a myriad of compounds, no matter whether a single herb medicine or a Chinese herbal formula, TCMs all need a unique methodology system to evaluate their quality. Characterized by multiple targets, components and links, TCM is based on a holistic philosophy that is totally different from Western medical practices. All these factors have led to the application of fingerprints in TCM research.

### 2.1. The Fingerprint

Obtained by using several techniques, both chromatographic and spectroscopic, a fingerprint is a characteristic pattern or profile which reflects the chemical constituents and usually represents as much information as possible [5,6]. Determining a few active ingredients or marker compounds to control the quality of TCM is far from sufficient. The quality control methods adopted for chemical medicines are generally not applicable to TCMs, because they lack a comprehensive evaluation of overall quality [7,8]. Moreover, the nature and amounts of chemical constituents in TCMs can be affected by many factors such as differences in harvest seasons, plant origins, processing methods and others [3]. For those reasons, any differences in the abovementioned factors can lead to different efficacies for a particular herbal medicine [9,10]. With the help of fingerprints, those variables can be well monitored and controlled.

TCM fingerprints can reflect the type and content of their chemical composition. Thus, they give an overall description and evaluation of herb quality. At present, the herbal medicine fingerprint technique is considered a powerful tool for the quality control of multi-component TCMs. Moreover, it has also internationally accepted as a feasible way to evaluate and control the quality of TCMs and their preparations around the world. Japanese scientists take fingerprints obtained from standard extraction methods as the standard fingerprint and the formula decoctions made from verified medicinal sources are regarded as the standard extract. Fingerprints have also started to be accepted by the US Food and Drug Administration, because the fingerprint method can be utilized in named material chemistry, manufacture and control (CMC) applications of Investigational New Drugs (INDs) to control the quality of Botanical Drug Substances and Botanical Drug Products. Besides, many countries and organizations such as Britain, France, Germany, India and the WHO have used fingerprinting to control the quality of medicinal plants. It has also been allowed and accepted by the Chinese State Food and Drug Administration (SFDA) for monitoring and risk management of TCM drug safety.

### 2.2. The Spectrum-Effect Relationship 

Using the chemical fingerprint to evaluate a TCM’s pros or cons obviously has some limitations. Some components embodied by fingerprints are not necessarily the efficacious ingredients, and the connection between the chemical composition and efficacy is thus unclear. Moreover, different preparation methods and different analysis methods or conditions may afford different fingerprints. Therefore, the fingerprint method was far from being a comprehensive way to control the quality of TCMs, because it cannot describe the authentic and general active pharmacological constituent information [2]. In 2001, Xie put forward the concept of development of TCM fingerprints, whereby fingerprints can be related to curative effects by just using chemical constituent information to control quality. Li *et al*. were the first to clearly propose the notion of the systematic chromatographic-pharmacodynamics relationships of TCMs in 2002. They first proposed the concept and called it “spectrum-effect relationship” [2]. The relevant mathematical model was being developed simultaneously, as chemometrics were first applied by Liang to “chromatographic-pharmacodynamics” for their practical significance, providing a basis for controlling the quality and evaluating the effects of TCMs.

“Spectrum-effect relationship” thus refers to linking the TCM fingerprint peaks with specific pharmacodynamic data to form a relationship, and using this relationship to look for the effective material(s) in Chinese medicines and to help formulate control standards to reflect its internal quality. To summarize, this Introduction has given details of the following topics: 

I. The fingerprint only considers the known chemical composition qualitatively or quantitatively, while the efficacy is not taken into account. Only on the premise of the curative effect of drugs, can the characteristic chemicals of a TCM fingerprint be related to its biological activity to better reflect the quality of the TCM. “Spectrum-efficiency relationships” may provide the possibility to compensate for the abovementioned shortcoming of fingerprints.

II. Through the analytic statistics method, “spectrum-efficiency relationships” could establish a mathematical model of a TCM’s chemical composition and efficacy; the “spectrum-effect relationship” technique uses informatics methods to find the alignment of key active compounds; it optimizes the compatibility of TCMs through the forecasting model of multi-component effects, and then builds the multi-factors regulating a network model of therapeutic effects; it finally explores the synergy mechanisms of the variety of aligned TCM active compounds. 

**Figure 2:**
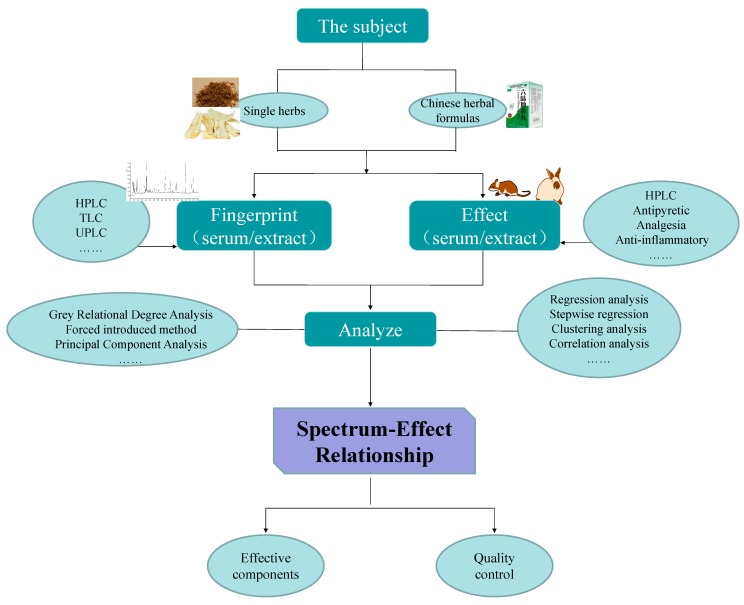
The general content of spectrum-effect relationship studies.

III. The changes in the peaks and their areas in established TCM fingerprints reflect differences in each component and its content; the pharmacodynamic model and indexes are chosen carefully so as to evaluate the pharmacological activity of the components more precisely; then using data processing technology we can analyze the weight coefficients of the components contributing to the observed efficacy; data processing methods have their respective pertinence, and various mathematical processing methods used in combination can complement each other and improve the precision and accuracy; then effective ingredient groups with synergism or antagonism effects can be sought out to further reveal the TCM effective material basis and establish the relationship between the spectrum and efficiency of TCM. The general content of spectrum-effect relationship studies can be summarized as shown in Figure 2.

IV Being an interdisciplinary and cutting-edge science, the spectrum-effect relationship is technology that integrates the Chinese medicine chemistry, analytical chemistry, pharmacodynamics and chemometrics of a TCM, so it is considered to be more accurate to study TCM active materials. Based on Figure 2, the research objects, methods of establishing fingerprints, pharmacodynamics studies and data processing methods of spectrum-effect relationships will be introduced in the following sections.

## 3. The Research Objects of the Spectrum-Effect Relationship Method

**Figure 3:**
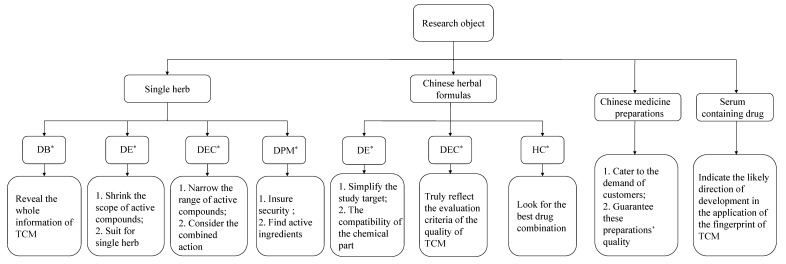
The spectrum-effect relationship research objects.

*Abbreviation*: DB—different batches; DE—different extracts; DEC—different extracts combination; DPM—different processing ways; HC—herbs combination.

Based on the habits of TCM drug usage, the research objects of spectrum-effect relationships may contain a single herb (Chinese herbal medicine, Chinese medicine decoctions) or a Chinese herbal formula. In addition, Chinese herbal preparations and sera containing drugs are two special study forms requiring special focus. In order to simplify the study, researchers use some different and appropriate processing approaches for each of them. The basic hierarchical chart of the research objects is shown in Figure 3.

### 3.1. Single Herbs

In Chinese medicine, a single herb has an unmatched effect. It can cure disease by itself or with the help of other herbs. For a feasible intricate interaction of different compounds, a single herb study would be a good start. Different ways of processing a single herb were done to reduce the degree of study difficulty. Through investigating the literature, we found that most related work is about the spectrum-effect relationships of single herbs (Table 1). It is divided into four objects under the caption “single herb”, that is different batches (DB), different extracts (DE), different extract combinations (DEC), and different processing methods (DPM), respectively.

DB refers to a single herb from a different production area, resource and collection time. DE comes from a single herb and different parts of one herb. Their extractive solvents may have different polarity. Their combination, sometimes with an orthogonal method, namely DEC, was also investigated.

Through DPW, different Chinese medicines showing different effects could be prepared from one kind of herb. This process is called “Paozhi”. It refers to different processing methods such as water treatment, fire treatment, both water and fire treatment and other ways. DPWs have a certain impact on the efficacy and toxicity. In standard TCM use practice, DPWs applied to the same Chinese herbal medicine may affect different symptoms and the toxicity will vary, so what makes this effect change is also a research hotspot. 

### 3.2. Chinese Herbal Formulas

Chinese herbal formulas, consisting of various single herbs, are the most common form used in clinical applications. Compared with single herbs, they possess unparalleled advantages when faced with miscellaneous diseases. The correlative research is relatively rare (Table 2), although TCM formulas are another main research object of the spectrum-effect relationship field. 

**Table 1:** Summary of single herb spectrum-effect relationships.

Chinese Herb Medicine	Processing Method	Fingerprint	Effects	Experimental Model	Analytical Method
*Vaccinium ashei* Reade	DB	HPLC	Antioxidant effect	Scavenge DPPH radical	HCA [11];
*Bostaurus domesticus* Gmelin.	DB DE	UPLC	Antibacterial effect	*Escherichia coli*	HCA, OMLR, PCA [12,13];
*Isatis* *indigotica* Fort.	DB	HPLC	Antibacterial effect	*Escherichia coli*	HCA, PCA, OMLR [14,15];
			Anti-endotoxin effect	Endotoxin	OMLR [16];
			Antibacterial effect	*Staphylococcus aureus*	HCA [17];
			Antivirus effect	Hemagglutination test	PCA [18];
*Psoralea corylifolia* Linn.	DB	HPLC	Antioxidant effect	Scavenge DPPH radical	CA [19];
*Angelica sinensis* (Oliv.) Diles	DB DPM	HPLC	Reinforcing Qi, Replenishing blood Scavenging free radicals	Mice Fenton reaction	GRDA [20]; OMLR [21];
*Cureuma* *kwangsiensis* S. G. Lee et C. F. Liang	DB	GC	Antitumor effect	Nasopharygeal carcinoma cells	GRDA [22];
*C*.*kwangsiensis* S. G. Lee et C. F. Liang stir-baked with vinegar	DB	HPLC	Dispersing blood stasis effect	Mice	GRDA [23];
*Polygoni* *cuspidati* Sieb. et Zucc.	DB DE	HPLC	Antibacterial effect Anticancer effect	Scavenge DPPH radical Mice, Rat, Leukemia cell line K 562, Lung cancer cell line A 549	BCA [24] PLSR, GRDA [25,26,27,28,29,30];
*Morina nepalensis* D. Don var. *alba* (Hand. -Mazz.) Y. C. Tang	DB	HPLC	Anti-inflammatory effect	RAW 264.7 cells	PLSR [31];
*Juglans* *mandshurica*	DE	TLC	Antitumor effect	BGC 803 cancer cells	CA [32];
*Paeoniae lactiflora* Pall.	DE	HPLC	Cooling blood effect	Rat alveolar macrophage NR 8383	PLSR [33,34];
*Rheum palmatum* L.	DB DPM	UPLC HPLC	Anti-HIV-1 effect Hemostatic effect	HIV-1 reverse transcriptase Mice	PCA [35]; GRDA [36];
*Rheum officinale* Baill.	DE	HPLC	Tyrosinase inhibitor	Tyrosinase	CA [37];
*Salvia miltiorrhiza* Bunge	DB DE	HPLC	Anti-oxidation effect	Fenton reaction Mice	BCA [38]; PLSR [39];
*Erigeron breviscapus* (Vant.) Hand-Mazz.	DEC	HPLC	Neuroprotective effects	SH-SY5Y cells	BCA [40];
*Euphorbia humifusa* Willd.	DE	HPLC	Antifungal effect	NCCLS M 38-A	GRDA [41];
*Cordyceps sinesis* (Berk.) Sacc.	DB	HPLC	Anti-hepatic fibrosis effect	LX-2 hepatic stellate cells	BCA [42];
*Alpinia officinarum* Hance.	DE	HPLC	Promotion of melanogenesis effect	Melanoma B 16 cells	GRDA [43];
*Pogostemon cahlin* (Blanco) Benth.	DE	HPLC	Anti-gastrointestinal propulsion effect	Mice	GRDA [44];
*Polygonum orientale* L.	DEC	UPLC	Protective effect on myocardial cells	Myocardial cells	BCA [45];
*Coptis chinensis* Franch.	DB DPM	HPLC UPLC	Ameliorating insulin resistanc Antibacterial effect	3T3-L1 preadipocyte ATP bioluminescence	PCA,CA,GRDA [46,47]; HCA [48]; OMLR [49];
*Astragalus membranaceus* (Fisch.) Bge. var. *mongholicus* (Bge.) Hsiao	DB DEC	HPLC	Improving immunity effect Anti-gastric ulcer effect Diuretic effect Antifatigue effect	Mice	GRDA [50]; PLSR,GRDA [41,42,43,44,45,46,47,48,49,50,51,52,53]; GRDA [54];
*Scutellaria baicalensis* Georgi	DB	HPLC	Antipyretic effect Antibaterial effect	Rat *Staphylococcus aureus*	GRDA [55]; GRNN [56];
*Bupleurum chinense* DC.	DE	HPLC	Hepatoprotective effect	Mice	HCA, TCA [57];
*Tinospora sagittata* (Oliv.) Gagnep.	DB DE	HPLC	Anti-inflammatory effect Analgesia effect	Mice	CA [58];
*Lonicera japonica* Thunb.	DB DE	HPLC	Anti-influenza virus effect Anti-inflammatory effect; Analgesia effect	MDCK cells Mice	OMLR [59,60];
*Murraya exotica* L.	DE	HPLC	Anti-inflammatory effect	Mice	GRDA [61];
*Zanthoxylum nitidum* (Roxb.) DC.	DB	IR	Antitumor effect Antineoplastic effect	7901, Hela cells	OMLR [62,63,64]
*Kalimeris indica* (L.) Sch-Bip	DB	HPLC	Anti-inflammatory effect	Mice	GRDA [65]
*Citrus grandis* (L.) Osbeck	DE	HPL C	Antioxidant effect	KMnO_4_	GRDA, CA, GRNN [66];
*Paeonia suffruticosa* Andr.	DE	HPLC	Promote blood circulation Remove blood stasis	Mice	OMLR [67];
*Cnidium monnieri* L.Cuss.	DE	HPLC	Sedative-hypnotic effect	Mice	CA [68];
*Artemisia capillaries* Thunb.	DE	HPLC	Hepatoprotective effect	Mice	CA [69];
*Crataegus pinnatifida* Bge.	DB	HPLC	Antioxidant effect	Rat	CA [70];
*Peucedunum harrysmithii* var. *subglabrum* (shan et sheh)	DB	HPLC	Eliminate phlegm effect	Mice	GRDA [71];
*Evodia ruatecarpa* (Juss.) Bneht. Var. *bodinieri* (Dode) Huang	DE	HPLC	Alleviate intestinal cramps effect	Rabbit	CA [72];
*Pseudostellaria heterophylla* (Miq.) Pax	DE	HPLC	Cytotoxic effect	MGC 80-3, RKO, HepG2 cells	CA [73];
*Panax notoginseng* (Burk.) F.H.Chen	DB	HPLC	Anti-myocardial ischemia effect	Rat	CA, FMA, PCA [74];
*Aconitum carmichaelii* Debx.	DPM	UPLC	Mitochondria growth promoting effect	Rat	CA [75];
*Aconitum* L.	DB	UPLC	Antibacterial effect	*Escherichia coli*	CA [76];

*Abbreviations*: DB—different batches; DE—different extracts; DEC—different extracts combination; DPM—different processing methods; HCA—Hierarchical Cluster Analysis; PLSR—Partial Least Squares Regression; OMLR—Ordinary Multiple Linear Regression; CA—Correlation Analysis; GRDA—Gray Regression Degree Analysis; FMA—Fuzzy Mathematical Analysis; PCA—The Primary Component Analysis; BCA—Bivariate Correlation Analysis; GRNN—General Regression Neural Network.

**Table 2:** Summary of Chinese herbal formula spectrum-effect relationships (for abbreviations refer to the footnote in Table 1).

Names	Involved TCMs	Fingerprint	Effects	Experimental Model	Analytical Method
Baihu Tang	*Anemarrhena* *asphodeloides* Bge. Gypsum fibrosum *Glycyrrhiza uralensis* Fisch. Rice	HPLC	Anti-inflammatory effect	Rat	BCA [77]
Danggui Chuanxiong	*A*. *sinensis* (Oliv.) Diels. *Ligusticum chuanxiong* Hort.	HPLC	Anti-myocardial ischaemia effect	Rat	BCA, OMLR [78]
Mongolian Preparation Sendeng-4 Decoction	*Xanthoceras sorbifoliae* Bunge. *Melia toosendan* Sieb.et Zucc *Terminalia chebula* Retz. *Gardenia jasminoides* Ellis.	HPLC	Anti-inflammatory and analgesic effect	Mice	OMLR [79]
Compound Wuren chun Capsules	*Schisandra chinensis* (Turcz.) Baill. *B*. *chinense* DC. *P*.*notoginseng* (Burk.) F. H. Chen *Phyllanthus urinaria* L.	HPLC	Liver protection	Rat	CA [80]
Gushu Dan	*Epimedium brevicornu* Maxim. *Drynaria fortunei*(Kunze) J. Sm. *C*. *monnieri* (L.) Cuss. *S*. *miltiorrhiza* Bge.	HPLC	The proliferative effect of osteoblast-like cells	Osteoblast-like cells	BCA, OMLR [81]
Tongsaimai Pellet	*A*. *membranaceus* (Fisch.) Bge.var.*mongholicus* (Bge.) Hsiao *G*. *uralensis* Fisch. *Lonicerae japonicae* Thunb. *Scrophularia ningpoensis* Hemsl. *Dendrobium nobile* Lindl. *A*. *sinensis* (Oliv.) Diels.	UPLC	Brain protection Vasodilatation effect PC12 cell injury protection;	Rat, Rabbit, PC 12 cells	HCA [82]
Xiaoyao Wan	*B*. *chinense* DC. *G*. *uralensis* Fisch. *A*. *sinensis* (Oliv.) Diels. *Paeonia lactiflora* Pall. *Atractylodes macrocephala* Koidz. *Poria cocos* (Schw.) Wolf *Mentha haplocalyx* Briq. *Zingiber officinale* Rosc.	HPLC GC	Anti-tyrosinase effect Anti-depression effect	B 16 melanoma cells Mice	BCA [83,84]
Jia Wei Si Miao Decoction	*Atractylodes lancea* (Thunb.) DC. *Phellodendron chinense* Schneid. *Achyranthes bidentata* Blume *Coix lacryma-jobi* L. var. *mayuen.* (Roman.) Stapf	GC HPLC	Anti-inflammatory effect Analgesia effect Decrease blood uric acid	Mice	OMLR, CA [85,86]
Ling Gui Shu Gan Tang	*P*. *cocos* (Schw.) Wolf *Cinnamomum cassia* Presl *A*. *macrocephala* Koidz. *G*. *uralensis* Fisch.	HPLC	Diuretic effect Anti-hypoxic effect	Mice	OMLR [87]
Shaoyao Gancao formulas	*P*. *lactiflora* Pall. *G*. *uralensis* Fisch.	HPLC	Analgesic effect	Mice	CA [88]
Qi Zhi Wei Tong	*B*. *chinense* DC. *Corydalis yanhusuo* W.T.Wang *Citrus aurantium* L. *Cyperus rotundus* L. *P*. *lactiflora* Pall. *G*. *uralensis* Fisch.	HPLC	Anti-inflammatory effect	RAW 264. 7 cells	GRDA, GRNN [89]
Sheng Hua Tang	*A*. *sinensis* (Oliv.) Diels *L*. *chuanxiong* Hort. *Prunus persica* (L.) Batsch *Zingiber offcinale* Rosc. *G*. *uralensis* Fisch.	HPLC	Invigorate the circulation of Qi	Rat	CA [90]
Tao Hong Si Wu Tang	*P*. *persica* (L.) Batsch *Carthamus tinctorius* L. *A*. *sinensis* (Oliv.) Diels. *Paeonia veitchii* Lynch *Rehmannia glutinosa* Libosch. *L*. *chuanxiong* Hort.	GC	Analgesic effect	Mice	OMLR, CA [91]
Wu Zhu Yu Tang	*Evodia rutaecarpa* (Juss.) Benth *Panax ginseng* C. A. Mey. *Z*. *officinale* Rosc. *Ziziphus zizyphus* Mill.	HPLC	Analgesic effect Anti-nausea effect	Mice	OMLR [92]
Xie Bai San	*Morus alba* L. *Lycium chinesnse* Mill. *G*. *uralensis* Fisch.	HPLC	Anti-inflammatory effect Expectorant effect	Mice	OMLR, BCA [93]
Zuo Jin Wan	*C*. *chinensis* Franch. *E*. *rutaecarpa* (Juss.) Benth.	HPLC	Biothermo-logical effect	*Escherichia coli*	CA [94]
Da Cheng Qi Tang	*R*. *palmatum* L. *Magnolia officinalis* Rehd. et Wils. *C*. *aurantium* L.	HPLC	Purgative effect	Mice	HCA [95]

For the meaning of abbreviations refer to the footnotes in Table 1.

The reason is mainly that the compounds in TCMs are so complicated and it is not easy to find out the component(s) related to a particular effect. Here, Chinese herbal formulas are divided into three objects: DE, DEC and herb combinations (HCs).

HC refers to a herb and its contents in an orthogonal formula combination. It can look for the best drug combination to display a synergy or attenuation effect. It also probes into the active substance or the main drugs in a formula. One way is considering the pharmacodynamic effect of a total formula, then removing a single drug or a group of drugs from the formula to measure the effect of the remaining part, and thus evaluate the removed drugs’ influence on the effect of the total formula. This method is applied to study the interaction of a single herb in a whole formula. Chen *et al*. used this method to analyze Sheng Hua Tang. They found that the total formula was superior to the decomposed recipe, and 13 compounds were identified as the likely material basis of the activity [90].

Another method is on the basis of the efficacy of a whole formula, doing parallel experiments using the same dose or different doses of each herb, or dividing them into several groups in accordance with the principle of “jun, chen, zuo, shi” or “medicine pairs” to do parallel experiments. Through the study of the dose variation of each herb in Wu Zhu Yu Tang, Ning *et al.* identified four components as its material basis [92]. 

### 3.3. Chinese Medicine Preparations

With the development of Chinese medicine, many Chinese medicine preparations such as granules, capsules, pills and injections are now produced in order to cater to the demands of customers. To guarantee these preparations’ quality, fingerprints have been widely recognized around the world as a holistic system because they provide the possibility for the control of TCM quality. Fingerprints are based on the characteristics of systemacity and stability and they determine a few active ingredients or marker compounds. This is far from enough to control the quality of TCMs, and the chemical medicine control mode is not suitable for TCMs without a comprehensive evaluation of overall quality. Spectrum-effect relationship studies would be a better choice. For instance, in the spectrum-effect relationship study of *Houttuynia cordata* injections, Lu *et al.* preliminarily solved the problem of its quality evaluation [96]. Related reports on preparations and sera are scarce, as shown in Table 3. 

### 3.4. Drug-Containing Serum 

Serum fingerprints, being a research hotpot since Shinichi [97] put forward the idea of serum- containing drugs in 1988, could clarify the real components that produce the healing efficacy *in vivo*. However, their complexity has hindered development to some extent.

TCMs contain many ingredients, and only the ingredients absorbed into the bloodstream can produce an effect. That is to say, the “transitional ingredients” in serum are likely to be the real basis of efficacy. Thus, by analyzing the components present in serum after oral administration, it is able to quickly and accurately determine the direct effect(s) the compounds are causing.

**Table 3:** Summary of the spectrum-effect relationships of Chinese medicine preparations and drug-containing sera.

Names	Type	Fingerprint	Effects	Experimental Model	Analytical Method
*Radix Astragali* Injection	Chinese medicine preparation	HPLC	Antioxidant effect	Scavenge DPPH radical	PLSR [98];
San Huang Preparation	Chinese medicine preparation	HPLC	Improve insulin resistance Antiendotoxin effect	Rat, The 3T3-L1 preadipocytes cells	OMLR, BCA, PCA [49,99];
Xiang Dan Injection	Chinese medicine preparation	HPLC	Anti-myocardial ischemia effect	Rat	GRNN [100];
Bu Zhong Yi Qi Wan	Serum containing drug	HPLC	Blood enriching effect	Mice	GRDA [101];
Xiao Yao Fang	Chinese medicine preparation	HPLC	Anti-depression effect	Rat	GRDA [102];
*H*. *cordata* Injection	Chinese medicine preparation	GC	Anti-inflammatory effect	Rat, Mice	HCA [96];
*Carthamus tinctorius* L.	Serum containing drug	CE	Increase the coronary artery flow Enhance the heart stroke amplitude Decrease the heart rate	Rabbit Guinea pig	CA [103].

For the meaning of abbreviations refer to the footnotes in Table 1.

Due to the fact that the regularity of distribution, absorption, metabolism and excretion of various components in the compound are different; the variation of the characteristic peaks in the corresponding fingerprints will be diverse. This provides a good entry point to understand the changes of complex components in the body. Serum fingerprints have been reported [104]. With the help advanced technology, such as UPLC-MS, it provides the possibility for studying spectrum-effect relationships.

Seen from the introduction of the above four research objectives, the efficacy of TCMs comes from a variety of active ingredients acting in synergy, or even the collaborative or antagonistic effects of some inactive ingredient(s), other than simply a single active chemical ingredient. Nowadays, some researchers only study the spectrum-effect relationships of different single fractions without considering their superposition. We suggest that establishing a continuous dynamic fingerprint with different polarity combinations can readily find the above functional components.

## 4. Methods for Establishing Fingerprints for Spectrum-Effect Relationships

There are a variety of methods to establish TCM fingerprints, including thin layer chromatography (TLC), high-performance liquid chromatography (HPLC), gas chromatography (GC), high speed counter current chromatography (HSCCC), mass spectroscopy (MS) and capillary electrophoresis (CE) [105]. Nowadays, in spectrum-effect relationship studies, almost only HPLC (or UPLC) and GC methods are used, and only three references to the use of CE, TLC and IR were found [32,36,62]).

When studying the spectrum-effect relationship, the fingerprints generally come from two sources: the chemical fingerprint of the TCM and the serum fingerprint of the TCM. Currently the most used method to establish fingerprints is HPLC, which offers a high degree of separation. A complex chemical composition can be easily separated into high or low peaks forming a chromatogram, but because of the high sensitivity, choosing the appropriate peaks always involves technical difficulties that can be hard to conquer. HPLC is mainly used to identify the corresponding ingredients of the fingerprint peaks and can provide a lot of information. It is also limited by the available detectors, for example the UV detector can only discern compounds having conjugated structures, and ELSD is for more volatile components only.

GC, with its high separation efficiency, high sensitivity, small amount of sample, *etc.*, is used to study the spectrum activity relationships of Chinese medicines containing volatile components*.* On the other hand GC is difficult to use in the analysis of thermally unstable substances and large molecular weight components, so its applications have been limited in spectrum-activity relationship studies.

Different methods for establishing fingerprints are suitable for different samples. Besides, when the relationship between a continuous dynamic fingerprint and the related effect(s) is established, common peaks, characteristic peaks with high content and the peaks whose content is changing in the fingerprint should all be chosen.

## 5. Pharmacodynamics Studies for Spectrum-Effect Relationships

When performing spectrum-effect relationship studies, usually there are two ways to obtain the effects. One is to choose a Chinese medicine extract to do pharmacodynamics studies, the other one is choosing serum containing the drug to do pharmacological experiments. Pharmacodynamics studies of Chinese medicines include *in vivo* and *in vitro* experiments. *In vivo* experiments use artificial animal pathology models to maintain the integrity of the body, ensuring normal contact with the external environment. Experimental studies can be carried while the animals are anesthetized or awake. *In vitro* experiments using organs or cells investigate the role of drugs on a particular portion of the overall body separately, so it is more intuitive [1]. We summarize the effects and pharmacodynamic models from the published articles about spectrum-effect relationships shown in Table 1, Table 2 and Table 3. Pharmacodynamic indexes and chosen models should follow the principle of reasonability, effectiveness and accuracy. 

### 5.1. The Pharmacodynamics Studies of TCM Extracts

In the pharmacodynamics studies of TCM extracts, after extraction and separation, the extract of the medicine was added directly to the reaction system or *in vitro* culture system to observe the effect. These experimental methods were carried out in *in vivo* and *in vitro* experiments.

There are some problems and defects in the methodology to do *in vitro* experiments directly using TCM crude extracts. This approach ignores that the vast majority of cases clinical administration of TCMs is through oral administration. After being metabolized by the gastrointestinal tract and liver, the components that finally reach the blood are usually not the original drug ingredients. There are also some metabolism ingredients. Besides, in the clinic TCM focuses on dialectical therapy. That is to say, TCM treats diseases in the case where certain syndromes exist (that is, a certain body state). It is hard to copy a disease which may be affected by other body systems. What’s more, TCMs consist of a variety of ingredients, thus the overall effect of the multi-component mixture is not equal to the effect of the various parts. Therefore, it is difficult to reflect the pharmacological effects and changing rules of the body in the real blood concentration.

### 5.2. Pharmacodynamics Studies of Drug-Containing Serum

Many ingredients in TCMs can only act after absorption into the bloodstream. The blood contained ingredients may be the substances playing a direct role in the body. Research on the components in the serum could be an effective approach to identify the effective substance(s). Using drug-containing serum instead of the decoction or crude extracts to do *in vitro* or *in vivo* experiments is thus also a research hotpot. 

The so-called drug-containing serum *in vitro* experiment refers to when after oral administration of a Chinese medicine or Chinese herbal compound to animals, a certain time later the animal’s blood is collected, the serum separated, and then this drug-containing serum is used to do pharmacological experiments. This can reflect not only the pharmacological effects of the components coming into the body and their metabolites, but also the role played by any drug-induced endogenous components. It can objectively simulate the process producing the pharmacological effects of the drug in an *in vivo* environment. The experimental conditions are closer to the internal environment where the drug plays is effects in the body, greatly improving the credibility of the experimental results.

We should base experiments on the clinical indications of Chinese medicine products to carry out pharmacodynamics studies at different levels of the whole body, organ, cell and molecular biology, to look for activity indicators which can more accurately reflect the observed effects. A TCM’s effect such as “strengthening the spleen”, “activating blood circulation to dissipate blood stasis”, “nourishing yin” and other effects are difficult to obtain or express by modern pharmacodynamic models. This needs further study [104]. 

## 6. Data Processing Methods of Spectrum-Effect Relationships

Data processing methods play a key role in “spectrum-effect relationship” studies. Different data processing methods will obviously affect the experiment results. For this reason, one or more data processing methods are used in combination when spectrum-effect relationships are established. A variety of methods each have their respective focus points. Combinations of multiple analysis methods and a secondary analysis of the data is the recommended pathway. Finally, the optimal spectrum-effect relationship will be obtained.

In recent years, scholars have been constantly improving the data processing methods applied in establishing the relationships. The processing methods used are not uniform, mainly including correlation analysis (CA), cluster analysis, regression analysis (mainly ordinary multiple linear regression, OMLR), gray correlation degree analysis (GRDA), fuzzy mathematical analysis (FMA), neural network analysis (GRNN) and the principal component analysis (PCA). According to the frequency of application, a simple introduction is shown as Table 4. Zhao *et al*. selected a Back Propagation Network (BPN) and Radial Basis Function Network (RBFN) to establish spectrum-activity relationship models of TCMs [106], and then used these models to predict efficacy and thus evaluate the quality of TCMs. Compared with the Hierarchical Cluster Analysis (HCA) and Partial Least Squares Regression (PLSR), the research results showed that the non-linear models based on neural networks are significantly better than the linear models, PCA and PLSR, whether it be in accuracy, prediction accuracy, or correlation coefficient. They were able to simulate well the complex nonlinear relationships between the various chemical components of the herbs and pharmacodynamic index of *L.* *chuanxiong* Hort., indicating the good application prospects for developing quality evaluation methods which can reflect the efficacy of TCMs in spectrum-effect relationship models. However, the data processing methods used have their respective defects, and one method alone is not always enough. Various mathematical processing methods using combined can be complement to each other, insure information maximization and improve accuracy. How to better combine them is another problem scholars should be delved into.

## 7. Conclusions and Future Perspectives

As a feasible approach to evaluate the effectiveness and safety of TCMs, spectrum-effect relationships has been considered for many years, though they are still at a preliminary stage, and this field has not yet formed mature and stable research ideas and methods.

There are some debatable issues in the existing research content [107]. We have also shown that there are some inconsistencies in the limited information presently available. The following measures should be applied to research of spectrum-effect relationships in the future:

**Table 4:** Common analytical methods applied in spectrum-effect relationships.

Method	Purpose	Advantages	Limits
CA	Study close degree between variable	Determine the relativity degree, significant extent and direction of change	Cannot explain the combined effect of the various peaks corresponding components to the pharmacodynamic indicators
HCA	Study the problem of classification, also known as group analysis	Intuitive, concise and achieve the classification	Cannot evaluate the correlation magnitude and the direction between fingerprint peaks and pharmacodynamic indicators
OMLR	Study a linear function to clarify the relationship between one dependent and two or more independent variables	Most commonly used method to study the intrinsic link	Not be able to see the contribution of peaks to the efficacy and not suitable for multiple correlation independent variables
PLSR	Allow the condition of the number of samples is less than that of variables to do regression modeling	Strong practicality and stability includes; Include all the original peaks of fingerprints	Abstract and difficult to understand; only suitable for qualitative analysis but not to determine the precise quantitative relationship between them
GRDA	Analyze the association degree of the various factors in system	Can use the known information to reveal unknown information	Difficult to describe overall contribution of the various peaks corresponding components through pharmacodynamic indicators
PCA	Elect fewer important variables from multiple variables through a linear transformation	Without loss of characteristic value number and information of sample	The amount of information after variable dimension reduction maintaining at a high level; The extracted principal component number being less than the original number of variables

For the meaning of abbreviations refer to the footnotes in Table 1.

### 7.1. The Application of Advanced Modern Analytical Technology

As shown in Figure 1, although research articles about spectrum-effect relationships represent a rising trend, most achievements were published in Chinese journals. This may mainly be because Western and traditional Chinese medical practices represent totally different philosophies, while spectrum-effect relationships are proposed based on a holistic concept. The fingerprint emphasizes the complete component spectrum rather than the exact nuances, and the information of quality it reflects is a comprehensive result. Current fingerprint technology can only evaluate the chemical constitution stability of different TCM batches, while lacking any judgment about their quality. Moreover, the amount of information reflected in a fingerprint can be too large, which is a major problem in spectrum-effect research. When the sample was not purified, due to the limitations of the detectors, each common peak in a HPLC trace may represent one or some mixed compounds, thus the established spectrum-effect relationship may not be accurate. Last but not the least, the purpose of the spectrum-effect relationship studies is to look for the active ingredients of TCMs. The current studies are focused on the active chromatographic peaks of the fingerprints, but there is no identification of the structures.

Therefore, the use of a variety of modern analytical technologies is especially necessary to identify the structures corresponding to the main peaks. The common modern analytical technology includes HPLC-MS, UPLC-MS, GC-MS, *etc.* Through the combination of multi-analytical technology, multi- fingerprints possessing various and complementary information can be established, so as to integrally express the overall chemical features of complicated material systems. By connecting the multi- fingerprints with effects, and seeking out the fingerprint peaks or component group related to the effects, an effect-fingerprint that can reflect the inherent quality is hopefully established, and thus we can evaluate the TCM quality by its chemical fingerprint. 

### 7.2. Selecting Pharmacodynamic Models Close to the Clinical Efficacy

Many westernized pharmacological models used nowadays in pharmacodynamics studies do not reflect the clinical efficacy of TCMs well. However, taking into account the needs for TCMs around the world, establishing appropriate models and indicators for evaluation of the efficacy of TCMs is an urgent problem that should be solved today [108]. When selecting a pharmacodynamic model, one should try to select a model close to Chinese medicine theory, so as to ensure the processed products screened according to the spectrum-activity relationships are able to display good efficacy in a clinical setting. From the different levels of the whole body, organs, cells and molecular biology, we should look for activity indicators which can more accurately reflect the effect and establish pharmacodynamic methods having *in vitro* and *in vivo* multi-indicators of TCM activity. The choice of a suitable pharmacodynamic index is also very important for the studies of multi-dimensional spectrum-effect relationships. 

## 7.3. Choosing the Proper Data Processing Method

At the present, data processing methods used in TCM research are not standardized, and a variety of data processing methods each have their particular focus. For this reason, it is crucial to select the appropriate data processing method in TCM research, but there are many difficulties in choosing a proper data processing method, because of the complexity of TCMs and data processing methods.

The various methods each have their respective strengths and weaknesses. One solution is to try to use them in combination, but different methods often produce different results because of the information loss, so further work should be done to establish more exclusive mathematical models, test their rationality, and use the model to identify chromatographic peaks that have positive and negative correlations with efficacy. Then enrich and prepare samples and proceed with structural identification, testing and verification of efficacy, and even identifying the active portion of a TCM instead of stopping at the point of the spectrum-effect relationship.

Spectrum-effect relationship studies are used to put forward new ideas and methods for exploring effective substances, optimizing formulas, improving preparation processes, tracking and separating target active ingredients, and for the development of new TCM drugs. It is actually interdisciplinary and cutting-edge science. More professional talents need to be introduced to the development of related software engineering. If people with different professional background contribute to TCM research by co-operating, there may be wider world waiting for us to explore. In short, the area of spectrum-effect relationships is a hot and difficult spot in the field of research on multi-component, multi-target, and multi-link TCMs, and it should be constantly addressed and perfected in practice.
